# Multicycle Autoclave Decontamination of N95 Filtering Facepiece Respirators

**DOI:** 10.1177/1535676020924171

**Published:** 2020-09-01

**Authors:** Nathen E. Bopp, Donald H. Bouyer, Christopher M. Gibbs, Joan E. Nichols, Corrie A. Ntiforo, Miguel A. Grimaldo

**Affiliations:** ^1^ Department of Pathology, University of Texas Medical Branch, Galveston, TX, USA; ^2^ Division of Infectious Disease, Department of Internal Medicine, University of Texas Medical Branch, Galveston, TX, USA; ^3^ Environmental Health and Safety, University of Texas Medical Branch, Galveston, TX, USA

**Keywords:** autoclave, decontamination, personal protective equipment, N95, surgical mask

## Abstract

**Introduction:**

During pandemic situations like the one caused by the emergent coronavirus SARS-CoV-2, healthcare systems face the challenge of limited personal protective equipment and impaired supply chains. This problem poses a threat to healthcare workers, first responders, and the public, which demands solutions that can span the gap between institutional shortages and resupplies.

**Objectives:**

To examine the efficacy of autoclave-based decontamination for the reuse of single-use surgical masks and N95 filtering facepiece respirators (FFRs). This method is the most readily available form of decontamination in the hospital and laboratory settings.

**Methods:**

Three models of N95 FFRs and two procedural masks were evaluated in this study. A moist heat autoclave using four different autoclave cycles: 115°C for one hour, 121.1°C for 30 minutes, 130°C for two minutes, and 130°C for four minutes was used. After the autoclave process, the FFRs were NIOSH fit tested and particle counting was performed for both coarse particles of 5 micrometers (µM) and fine particles from 0.1µM to 1.0µM.

**Results:**

We observed negligible alterations in the functionality and integrity of 3M 1805 and 3M 1870/1870+ N95 FFRs after three autoclave cycles. Surgical masks also showed minimal changes in functionality and integrity. The 3M 1860 FFR failed fit test after a single autoclave decontamination cycle.

**Discussion and Conclusion:**

The study finds that specific surgical masks and N95 FFR models can withstand autoclave decontamination for up to three cycles. Additionally, the autoclave cycles tested were those that could be readily achieved by both clinical and research institutions.

Medical grade filtering facepiece respirators (FFRs) are an essential form of personal protective equipment (PPE) that help to limit an individual’s exposure to pathogens transmitted by droplets and/or aerosols. Due to the emergence of severe acute respiratory syndrome coronavirus 2 (SARS-CoV-2) in late 2019 and the subsequent pandemic that it caused, a global increase in demand for PPE has occurred.^1-3^ Unfortunately, many countries have been unable to meet this demand and are forced to reuse PPE that was initially intended for single use. Several studies have previously been conducted to study the effects of various decontamination methods on PPE, with a focus on FFRs.^2,4-10^ These studies found that multiple decontamination methods can be used on N95 FFRs with varying effects on the functionality and integrity of the FFRs. Some of these methods, although effective, are labor-intensive and require specialized equipment and expertise to perform. In this study, we set out to test moist heat autoclave decontamination on N95 FFRs. This decontamination capability is available at many hospitals, outpatient clinics, and laboratories where it can be used with standardized training. Additionally, this method allows for rapid deployment and high throughput decontamination of FFRs. Several cycle strategies were tested, ranging from low temperatures for extended periods of time to high temperatures for short periods.

## Methods

### Masks

Three models of N95 FFRs were tested in this study, molded 3M 1860, folded 3M 1805, and folded 3M 1870/1870+ FFRs. Two different surgical face masks were used in this work: American Society for Testing and Materials (ASTM) level 1 procedural masks (Life Science Products) and ASTM level 2 procedural masks (Life Science Products).

### Autoclave Decontamination

**Figure 1.**
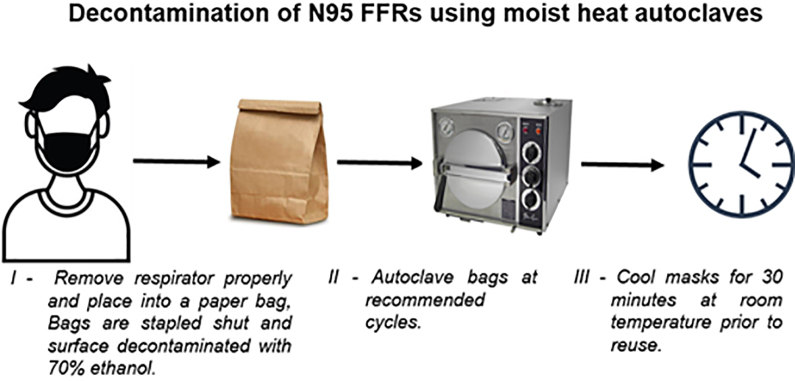
Representation of the decontamination workflow.

A moist heat autoclave was used for this study. The autoclave cycles were programmed to have 3 prevacuum stages at 1.5 pounds per square inch absolute (psia) each, a sterilization process at the trial exposure temperature and time, and then 10 minutes of drying time. Four autoclave cycles were examined in this study: 115°C for 1 hour,^11,12^ 121.1°C for 30 minutes, 130°C for 2 minutes, and 130°C for 4 minutes.^11,12^ These decontamination cycle parameters were selected not only to control the contamination with SARS-CoV-2 but also to account for potential contamination of the PPE by flora obtained from the clinical environment and/or the end-user. The decontamination cycles were validated using Comply SteriGage Steam Chemical Integrators (3M) and ATTEST Biological Indicator (3M) in the Super Rapid 5 Steam Plus Challenge Packs (3M). Generic kraft paper lunch bags and autoclavable pouches (Crosstex) were used to contain the FFRs and the masks during the decontamination process (Figure 1).

### Particle Counting

**Figure 2.**
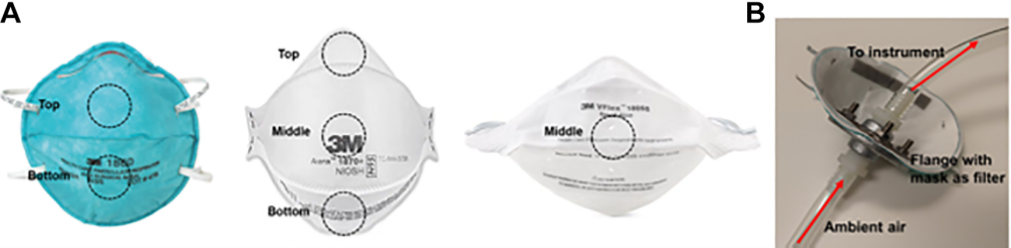
Forced particle penetration of N95 FFRs. Three types of 3M N95 FFRs were used in this study: model 1860, model 1805, and model 1870/1870+. (A) Regions of each FFR tested for particle penetration. (B) An example of a model 1860 mounted and connected to an Aerotek Portable Particle Counter. A total of 283 L of air was forced through each 1-inch-diameter test section of the mask.

Particle counting was performed using an Aerotek Portable Particle Counter model 9110 (TSI) with a modified flange setup. The testing procedure was designed to evaluate both coarse particles of 5 µm and fine particles from 0.1 µm to 1.0 μm, as established in previous studies.^13,14^ These sizes of particles are meant to represent pathogens in droplets (coarse particles) or aerosols (fine particles). Ten sampling replicates of 1 minute each at a flow rate of 28.3 L of air per minute were measured through a 6.4-cm^2^ round section of the filter media (Figure 2). Two sites were measured on the 3M 1860 FFRs, 1 site on the 3M 1805 FFRs, and 3 sites on the 3M 1870/1870+ FFRs. A single site was measured for each surgical mask. Particle count readings of the decontaminated FFRs and masks were compared with unexposed control samples. Calculations for the filtration efficiency determination were made using the average of the replicates and comparison of decontaminated samples against control samples.

### Fit Testing

Quantitative fit testing was performed on an individual using Portacount Respirator Fit Tester 8030 (TSI) following the National Institute for Occupational Safety and Health (NIOSH) requirements.^15^ Four exercises were used: bending over (50 seconds), talking (30 seconds), moving head side to side (30 seconds), and moving head up and down (30 seconds). A final fit factor score was calculated from the results of each category, and a final score of ≥100 was required for passing.

## Results

### Molded N95 FFRs

The 3M 1860 series did not pass fit testing under any of our decontamination conditions (Table 1). Observable deformation of the FFR was noticed at all temperatures and separation of layers was observed at higher temperatures. Although these FFRs did not pass the fit test, we continued to evaluate their ability to filter particulates. No significant change in the ability of the FFR to filter particles of 0.3 µm size and larger compared with its control was observed after a single moist steam decontamination cycle at 115°C for 60 minutes or 121°C for 30 minutes (Table 1). A minor change in filtration efficiency was observed after autoclave decontamination temperatures of 130°C for 2 minutes or 4 minutes (Table 2).

**Table 1.** Results of Moist Heat Autoclave Decontamination of 3M 1860 FFRs at 115°C for 60 Minutes and 121.1°C for 30 Minutes.^a^

Moist Steam Decontamination Method	Mask Type	SteriGage Result	ATTEST Result	NIOSH Fit Test Result	Mean Retention of Aerosolized Particles (%R) (n = 1)			
					0.3 μm	1.0 μm	5.0 μm	
Control	1860	NA	NA	Pass	98.62	99.66		99.89
115°C for 60 min (1 cycle)	1860 (n = 3)	Pass	Pass	Fail (3 of 3)	89.56	99.58		100
121°C for 30 min (1 cycle)	1860 (n = 3)	Pass	Pass	Fail (3 of 3)	88.55	98.78		99.96

^a^ NA indicates not applicable.

**Table 2.** Results of Moist Heat Autoclave Decontamination of 3M 1860 FFRs at 130°C for 2 Minutes and 130°C for 4 Minutes.^a^

Moist Steam Decontamination Method	Mask Type	SteriGage Result	ATTEST Result	NIOSH Fit Test Result	Mean Retention of Aerosolized Particles (%R) (n = 1)		
					0.3 μm	1.0 μm	5.0 μm
Control	1860	NA	NA	Pass	98.62	99.66	99.89
130°C for 2 min (1 cycle)	1860 (n = 3)	Pass	Pass	Fail (3 of 3)	87.05	98.55	99.89
130°C for 4 min (1 cycle)	1860 (n = 3)	Pass	Pass	Fail (3 of 3)	82.32	97.38	99.87

^a^ NA indicates not applicable.

### Folded N95 FFRs

**Figure 3.**
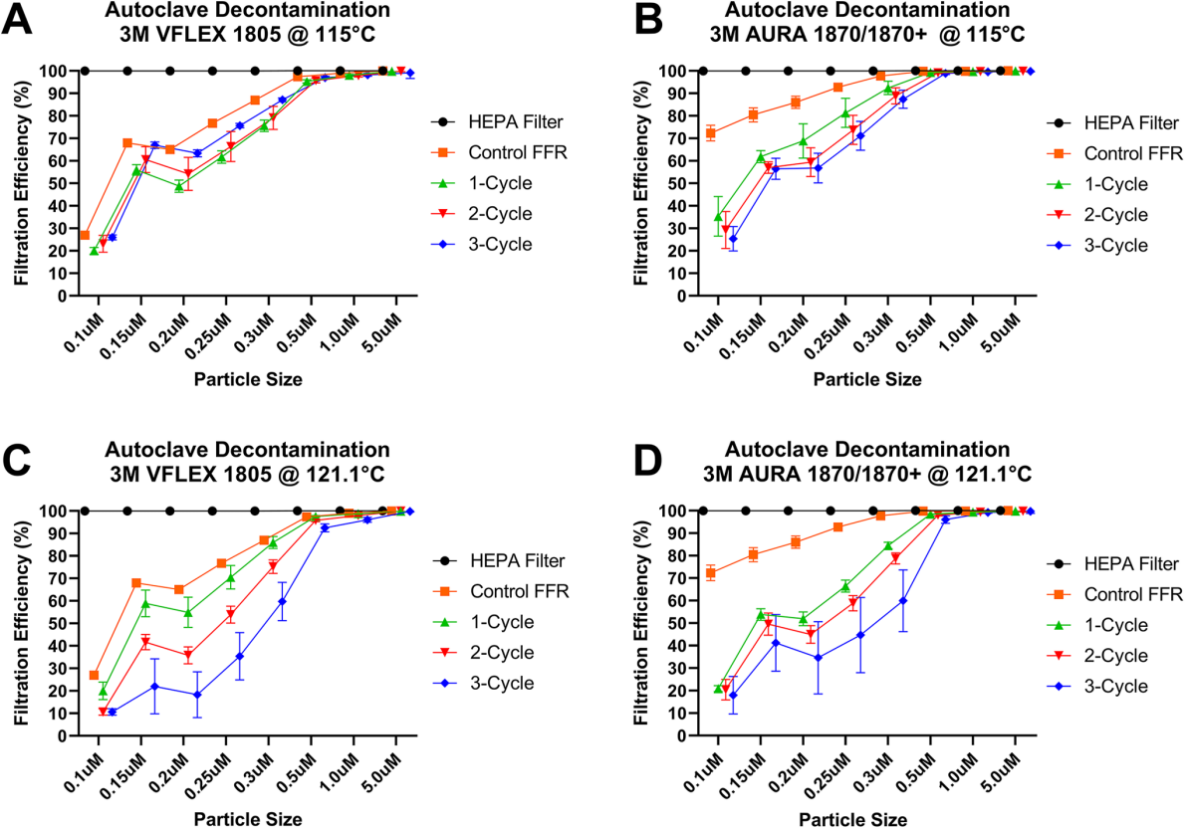
Filtration efficiency effects of autoclave decontamination of 3M N95 FFRs VFLEX 1805 and AURA 1870/1870+. (A) Filtration efficiency of VFLEX 1805 at 115°C. (B) Filtration efficiency of AURA 1870/1870+ at 115°C. (C) Filtration efficiency of VFLEX 1805 at 121.1°C. (D) Filtration efficiency of AURA 1870/1870+ at 121.1°C.

Folded FFRs were represented by the 3M 1805 and 3M 1870/1870+ models. The 3M 1805 FFRs passed fit testing for up to 3 decontamination cycles, at both 115°C and 121.1°C (Tables 3 and 4). The 3M 1870/1870+ FFR passed fit testing for up to 3 decontamination cycles at both 115°C and 121.1°C but began to fail at 5 cycles at 121.1 °C (Tables 5 and 6). No apparent visual deterioration of the filter material was observed, which was confirmed by only a slight reduction in particle retention (Tables 3 [Table table4-1535676020924171] [Table table5-1535676020924171]-6). A slight reduction of particle retention was noticed under the 121.1°C cycles compared with the 115°C decontamination cycles (Figure 3). Also, a weakening of the rubber bands was seen in the samples after 3 cycles.

**Table 3.** Results of Moist Heat Autoclave Decontamination of 3M 1805 FFRs at 115°C for 60 Minutes.^a^

Method	Mask Type	SteriGage Result	ATTEST Result	NIOSH Fit Test Result	Mean Retention of Aerosolized Particles (%R)		
					0.3 μm	1.0 μm	5.0 μm
Control	1805	NA	NA	Pass	86.96 ± 0.18	98.97 ± 0.13	99.92 ± 0.17
115°C for 60 min (1 cycle)	1805 (n = 3)	Pass	Pass	Pass (3 of 3)	75.58 ± 2.50	98.04 ± 0.45	99.81 ± 0.47
115°C for 60 min (2 cycles)	1805 (n = 3)	Pass	Pass	Pass (3 of 3)	79.08 ± 5.15	97.83 ± 0.54	99.88 ± 0.24
115°C for 60 min (3 cycles)	1805 (n = 3)	Pass	Pass	Pass (3 of 3)	87.09 ± 0.86	98.25 ± 0.64	99.16 ± 2.53

^a^ NA indicates not applicable.

**Table 4. table4-1535676020924171:** Results of Moist Heat Autoclave Decontamination of 3M 1805 FFRs at 121.1°C for 30 Minutes.^a^

Method	Mask Type	SteriGage Result	ATTEST Result	NIOSH Fit Test Result	Mean Retention of Aerosolized Particles (%R)		
					0.3 μm	1.0 μm	5.0 μm
Control	1805	NA	NA	Pass	86.96 ± 0.18	98.97 ± 0.13	99.92 ± 0.17
121.1°C for 30 min (1 cycle)	1805 (n = 3)	Pass	Pass	Pass (3 of 3)	86.96 ± 2.70	98.79 ± 0.24	99.91 ± 0.17
121.1°C for 30 min (2 cycles)	1805 (n = 3)	Pass	Pass	Pass (3 of 3)	75.21 ± 2.98	97.96 ± 0.39	99.93 ± 0.15
121.1°C for 30 min (3 cycles)	1805 (n = 3)	Pass	Pass	Pass (3 of 3)	59.71 ± 8.51	96.04 ± 1.05	99.83 ± 0.25

^a^ NA indicates not applicable.

**Table 5. table5-1535676020924171:** Results of Moist Heat Autoclave Decontamination of 3M 1870/1870+ FFRs at 115°C for 60 Minutes.^a^

Method	Mask Type	SteriGage Result	ATTEST Result	NIOSH Fit Test Result	Mean Retention of Aerosolized Particles (%R)		
					0.3 μm	1.0 μm	5.0 μm
Control	1870/1870+	NA	NA	Pass	97.71 ± 0.52	99.79 ± 0.09	100.0
115°C for 60 min (1 cycle)	1870/1870+ (n = 3)	Pass	Pass	Pass (3 of 3)	92.41 ± 2.95	99.69 ± 0.10	99.92 ± 0.22
115°C for 60 min (2 cycles)	1870/1870+ (n = 3)	Pass	Pass	Pass (3 of 3)	88.86 ± 3.58	99.72 ± 0.07	99.91 ± 0.23
115°C for 60 min (3 cycles)	1870/1870+ (n = 3)	Pass	Pass	Pass (3 of 3)	87.38 ± 3.98	99.55 ± 0.19	99.83 ± 0.32

^a^ NA indicates not applicable.

**Table 6.** Results of Moist Heat Autoclave Decontamination of 3M 1870/1870+ FFRs at 121.1°C for 30 Minutes.^a^

Method	Mask Type	SteriGage Result	ATTEST Result	NIOSH Fit Test Result	Mean Retention of Aerosolized Particles (%R)		
					0.3 μm	1.0 μm	5.0 μm
Control	1870/1870+	NA	NA	Pass	97.71 ± 0.52	99.79 ± 0.09	100.0
121.1°C for 30 min (1 cycle)	1870/1870+ (n = 5)	Pass	Pass	Pass (5 of 5)	84.48 ± 1.46	99.35 ± 0.08	99.76 ± 0.40
121.1°C for 30 min (2 cycles)	1870/1870+ (n = 5)	Pass	Pass	Pass (5 of 5)	78.76 ± 2.44	99.34 ± 0.19	99.75 ± 0.37
121.1°C for 30 min (3 cycles)	1870/1870+ (n = 5)	Pass	Pass	Pass (5 of 5)	59.92 ± 13.69	99.20 ± 0.30	99.69 ± 0.89
121.1°C for 30 min (5 cycles)	1870/1870+ (n = 3)	Pass	Pass	Fail (2 of 3)	NT	NT	NT

^a^ NA indicates not applicable; NT indicates not tested.

### Surgical Masks

Both ASTM level 1 and ASTM level 2 procedural masks underwent multiple rounds of autoclaving at 121°C for 30 minutes (Tables 7 and 8, respectively). No observable deformation occurred to the masks. Models with the attached face shields sustained damage to the shields, but the mask retained its filtering capacity. Masks showed minimal change in their ability to filter particulates ranging from 1 µm to 5 µm when compared with the control samples. Additionally, no loss in the strength of the straps was observed.

**Table 7.** Results of Moist Heat Autoclave Decontamination of ASTM Level 1 Procedural Mask at 121.1°C for 30 Minutes.^a^

Method	Mask Type	SteriGage Result	ATTEST Result	NIOSH Fit Test Result	Mean Retention of Aerosolized Particles (%R)		
					0.3 μm	1.0 μm	5.0 μm
Control	ASTM level 1 procedural mask	NA	NA	NA	40.86 ± 0.62	70.74 ± 0.90	98.22 ± 0.66
121.1°C for 30 min (3 cycles)	ASTM level 1 procedural mask (n = 3)	Pass	Pass	NA	42.84 ± 1.19	68.87 ± 3.51	96.99 ± 2.30

^a^ NA indicates not applicable.

**Table 8.** Results of Moist Heat Autoclave Decontamination of ASTM Level 2 Procedural Mask at 121.1°C for 30 Minutes.^a^

Method	Mask Type	SteriGage Result	ATTEST Result	NIOSH Fit Test Result	Mean Retention of Aerosolized Particles (%R)		
					0.3 μm	1.0 μm	5.0 μm
Control	ASTM level 2 procedural mask	NA	NA	NA	63.37 ± 0.23	96.86 ± 0.17	99.18 ± 1.46
121.1°C for 30 min (3 cycles)	ASTM level 2 procedural mask (n = 3)	Pass	Pass	NA	42.24 ± 0.88	93.25 ± 0.28	98.91 ± 1.02

^a^ NA indicates not applicable.

## Discussion and Conclusion

The primary function of a filtering facepiece respirator in the clinical or infectious disease research environment is to prevent the transfer or acquisition of viral or bacterial pathogens through contaminated droplet particles (≥5 μm) or aerosolized particles (≤5 μm). A recent study has shown that surgical masks can offer a level of protection against droplets containing coronaviruses, influenza, and, to a lesser extent, rhinoviruses.^14^ However, a lack of FFRs to use in high-risk situations can place healthcare workers, first responders, and researchers at risk. The objective of this study was to determine whether FFRs that had been subjected to autoclave decontamination could be recycled for use. We began our research with the hypothesis that lower temperatures in the autoclave process, using the method of F_0_ calculation, could reduce the damage to the filtering materials used to manufacture FFRs.^11,12^ We initially selected a decontamination cycle with a lower temperature for a longer time (115°C for 60 minutes) and a higher temperature for a short time interval (130°C for 2 or 4 minutes) versus the typical 121.1°C autoclave cycles. During the execution of the project, we discovered that the autoclaves in the healthcare setting could not readily be adjusted to the lower temperatures. Therefore, we had to concentrate our efforts to test the FFRs at 121.1°C (250°F).

In this study we used 2 different components when evaluating facepiece respirator function. One is the respirator fit test and the second is the functionality of the respirator to remove particles from the air a person is breathing. NIOSH requires an annual respirator fit test to confirm the fit the appropriate style, size, and model of any respirator to a wearer’s face. The fit test examines the seal between the respirator’s facepiece and the worker’s face. The respirator must fit the user’s face snugly (ie, create an appropriate seal) in order to minimize the number of particles that bypass the filter through gaps between the user’s skin and the respirator seal. The second component is the filtration function of the PPE, which needs to be highly effective at capturing particles that pass through the material that the respirator is composed of. Our choice of evaluating both fit test and filter effectivity allows us to consider both loss of “fit” following decontamination of masks and loss of function due to changes in the filtration of particles by each mask type or evaluation of filter degradation.

Our results indicate that under certain conditions, moist steam sterilization works to decontaminate certain models of N95 FFRs effectively. Each of the folded 3M 1805 and 1870/1870+ N95 models showed no apparent degradation to the mask or loss of fit testing ability after autoclave exposures of either 115°C for 60 minutes or 121°C for 30 minutes. We did observe a reduction in the retention of 0.3-µm particles in the FFRs with each sequential autoclave decontamination cycle. Even though particle retention was reduced after every autoclave cycle, the 1805 and 1870/1870+ FFRs complied with NIOSH fit testing standard requirements for up to 3 autoclave decontamination cycles.^15^ Our data indicate that the FFRs are still highly effective against droplet (coarse particle of ≥5 μm) contamination with levels close to 99% of that of the controls (Tables 3 [Table table4-1535676020924171] [Table table5-1535676020924171]-6). When analyzing the particle data for aerosols, our results again showed that all of the 3M folded FFRs tested were 99% effective in particle exclusion in comparison to the control (Tables 3 [Table table5-1535676020924171]-6). We noted a slight loss of elasticity in the rubber straps of the FFRs with each autoclave treatment. This is not surprising as the rubber straps are probably the most susceptible FFR component to all decontamination processes. This has also been observed by other groups when testing other decontamination methods such as vaporous hydrogen peroxide (Battelle report). The individuals who fit tested the 1805 and 1870/1870+ FFRs that underwent up to 3 cycles of decontamination did not perceive any differences in the ease of breathing in comparison to controls. However, 1 wearer did express difficulty breathing with the FFRs that had been decontaminated 5 times. In addition, the 5-decontamination cycle FFRs failed (2 out of 3) the NIOSH quantitative fit test.

Molded masks, such as the 3M 1860, failed fit testing after single-cycle autoclaving even though they did not have a significant decrease in 0.3-µm particle retention (Tables 1 and 2). Surgical masks were also tested during this study, and the data showed minimal changes in particle retention (Tables 7 and 8).

During times of national emergencies such as the pandemic caused by SARS-CoV-2, first responders, healthcare, and research institutions can be overwhelmed by the demands generated by such events.^3^ Resources can rapidly be depleted, and replacement material and supplies may be difficult to replenish. One of the significant issues caused by this pandemic is the difficulty seen by institutions in obtaining PPE. One method to address potential shortages in PPE is to examine the possibility of decontaminating PPE for reuse. In this study, we sought to determine whether autoclaving is a viable option for specific N95 models and procedural masks with a limited impact on their filtration capabilities. We observed that 3M 1870 and 1870+ FFRs and ASTM level 1 and level 2 procedural masks retained usability after 3 autoclave cycles under the conditions as mentioned above. Under none of the tested conditions did we observe molded FFRs (3M 1860) maintaining usability. These data suggest that this type of FFR requires a different decontamination method.
